# Genome-Wide Association Studies Reveal the Genetic Basis of Fertility Restoration of CMS-WA and CMS-HL in *xian*/*indica* and *aus* Accessions of Rice (*Oryza sativa* L.)

**DOI:** 10.1186/s12284-020-0372-0

**Published:** 2020-02-10

**Authors:** Pingbo Li, Hao Zhou, Hanyuan Yang, Duo Xia, Rongjia Liu, Ping Sun, Quanxiu Wang, Guanjun Gao, Qinglu Zhang, Gongwei Wang, Yuqing He

**Affiliations:** National Key Laboratory of Crop Genetic Improvement and National Center of Crop Molecular Breeding, Huazhong Agricultural University, Wuhan, 430070 China

**Keywords:** GWAS, *Rf* genes, CMS-WA, CMS-HL, Haplotype analysis, Rice

## Abstract

**Background:**

Wild-abortive cytoplasmic male sterility (CMS-WA) and Honglian CMS (CMS-HL) are the two main CMS types utilized in production of three-line hybrid rice in *xian*/*indica* (*XI*) rice. Dissection of the genetic basis of fertility restoration of CMS-WA and CMS-HL in the core germplasm population would provide valuable gene and material resources for development of three-line hybrid combinations.

**Results:**

In this study, two F_1_ populations with CMS-WA and CMS-HL background respectively were developed using 337 *XI* and *aus* accessions being paternal parents. Genome-wide association studies on three fertility-related traits of the two populations for two consecutive years revealed that both fertility restoration of CMS-WA and CMS-HL were controlled by a major locus and several minor loci respectively. The major locus for fertility restoration of CMS-WA was co-located with *Rf4*, and that for fertility restoration of CMS-HL was co-located with *Rf5*, which are cloned major *restorer of fertility* (*Rf*) genes. Furthermore, haplotype analysis of *Rf4*, *Rf5* and *Rf6*, the three cloned major *Rf* genes, were conducted using the 337 paternal accessions. Four main haplotypes were identified for *Rf4*, and displayed different subgroup preferences. Two main haplotypes were identified for *Rf5*, and the functional type was carried by the majority of paternal accessions. In addition, eight haplotypes were identified for *Rf6*.

**Conclusions:**

Haplotype analysis of three *Rf* genes, *Rf4*, *Rf5* and *Rf6*, could provide valuable sequence variations that can be utilized in marker-aided selection of corresponding genes in rice breeding. Meanwhile, fertility evaluation of 337 accessions under the background of CMS could provide material resources for development of maintainer lines and restorers.

## Background

Cytoplasmic male sterility (CMS) in higher plants is characterized by the inability to produce functional pollens, and is caused by chimeric open reading frame (ORF) in the mitochondrial genome. Nuclear-encoded *restorer of fertility* (*Rf*) genes produce proteins that are targeted to mitochondrial and can suppress the function of ORFs conferring CMS (Chen and Liu 2014). The exploitation of CMS and *Rf* genes systems in rice facilitate the development and commercialization of the three-line hybrid rice, which has made tremendous contribution to the food security worldwide (Li et al. 2007). According to that the pollen fertility is determined by the sporophytic genotype or gametophytic genotype, CMS could be divided into two types, sporophytic and gametophytic types. Wild-abortive CMS (CMS-WA) and Honglian CMS (CMS-HL) are the two main CMS types utilized in production of three-line hybrid rice in *xian*/*indica* (*XI*) rice, and CMS-WA is a typical sporophytic type while CMS-HL is a gametophytic type (Li et al. 2007). Mining and cloning of *Rf* genes for the two CMS types could be of great use in development of three-line hybrid rice combinations, and further improving the yield of rice.

Sterile lines carrying CMS-WA produce pollens that display irregular shape and no starch accumulation (Chen and Liu 2016). CMS-WA is caused by *WA352*, a novel mitochondrial gene originated recently in wild rice, and the protein WA352 abolishes the function of COX11 in peroxide metabolism, further triggers premature tapetal programmed cell death and finally results in the abortion of pollens (Luo et al. 2013). Previous studies showed that two major genes, *Rf3* and *Rf4*, could restore the fertility of CMS-WA (Yao et al. 1997, Zhang et al. 1997). *Rf4* encodes a pentatricopeptide repeat (PPR) protein with 782 amino-acid residues, which suppresses WA352-mediated male sterility by decreasing *WA352* mRNA levels (Kazama and Toriyama 2014, Tang et al. 2014). However, *Rf3* is still under cloning. In addition, several minor loci conferring fertility restoration of CMS-WA were reported (Bazrkar et al. 2008, Zhuang et al. 2001).

Sterile lines carrying CMS-HL produce pollens that display regular shape and no starch accumulation (Hu et al. 2016). CMS-HL is caused by the mitochondrial gene *orfH79*, and the protein ORFH79 interacts with a subunit of the mitochondrial electron transport chain complex III, further causes energy production dysfunction and oxidative stress, and finally leads to abnormal pollen development (Peng et al. 2010, Wang et al. 2013). Two major genes, *Rf5* and *Rf6*, could restore fertility of CMS-HL (Huang et al. 2012a). *Rf5* encodes a protein with 16 PPR motifs, which is a component of a restoration of fertility complex conferring the processing of CMS-associated transcript *atp6-orfH79*, together with at least another two members (Hu et al. 2012, Qin et al. 2016). Similarly, *Rf6* encodes a protein with 20 PPR motifs, and the protein RF6 forms a new complex with other members to cleave the aberrant transcript *atp6-orfH79* (Huang et al. 2015). However, the co-existence of *Rf5* and *Rf6* can only restore fertility of 75% of pollens. In order to further improve pollen fertility and seed-setting rate of F_1_ lines with CMS-HL, novel *Rf* genes are awaited to be identified.

Genome wide association studies (GWAS) have been proved to be powerful in dissection of complex traits in rice (Han and Huang 2013). Up to now, the genetic bases of many important agronomic traits of rice, such as flowering time, grain yield traits, tiller angle, panicle architecture and out-crossing traits, have been investigated with GWAS (Bai et al., 2016, Dong et al. 2016, Guo et al. 2017, Huang et al. 2012b). However, GWAS has not been applied to dissect the genetic basis underlying fertility restoration of CMS-WA and CMS-HL. Therefore, two F_1_ populations with CMS-WA and CMS-HL background respectively were developed using 337 *XI* and *aus* accessions being paternal parents in this study, and subjected to evaluation of three fertility-related traits. GWAS of the three traits revealed the corresponding genetic basis. In addition, haplotype analysis of *Rf4*, *Rf5* and *Rf6*, the three major genes for CMS-WA and CMS-HL respectively, were conducted.

## Results

### Variation and Correlation of Fertility-Related Traits

**Fig. 1:**
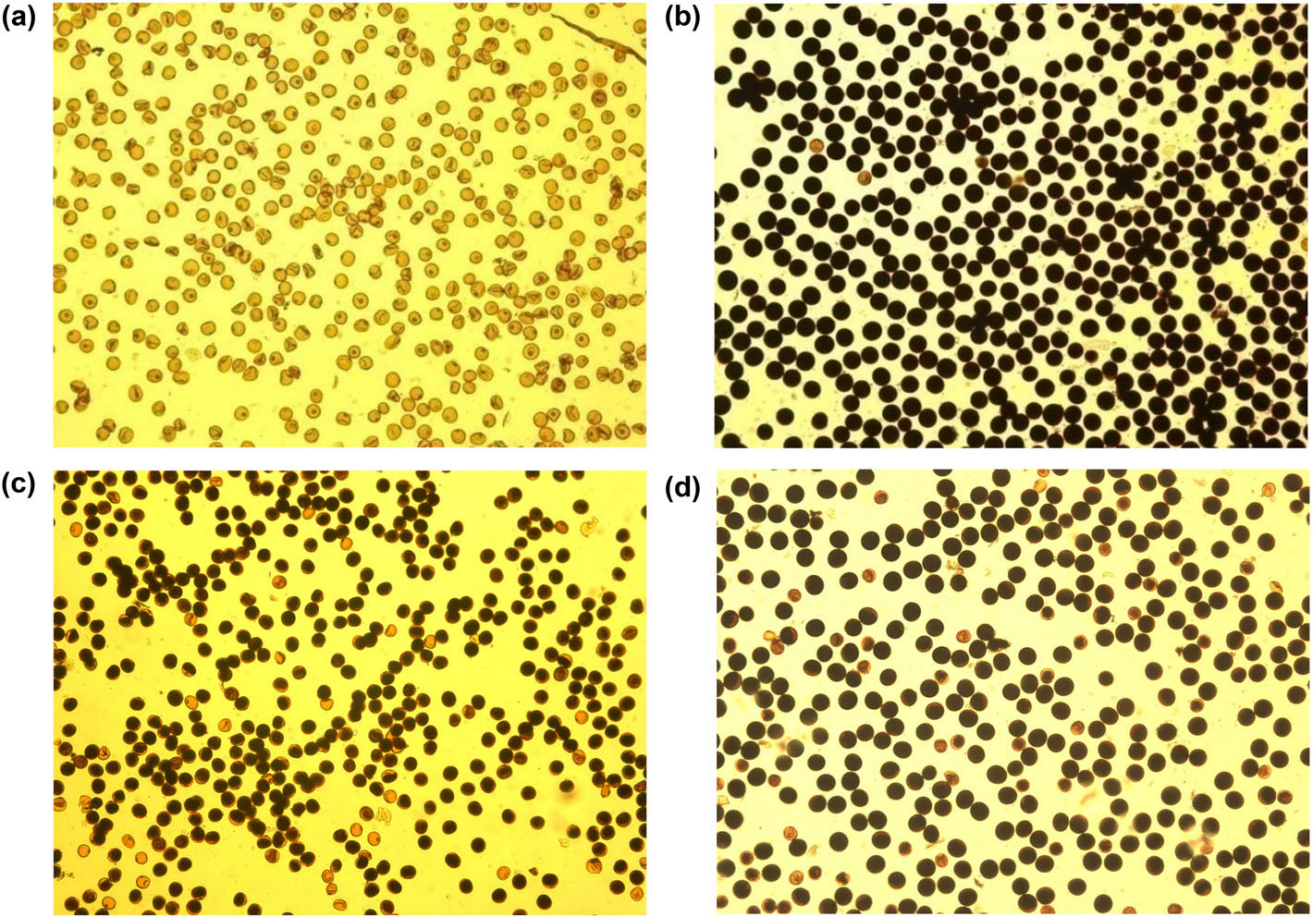
Pollens of HUA (**a**), HUA/MH63 (**b**), YTA (**c**) and YTA/9311 (**d**), stained with a 1% I_2_-KI solution

As shown in Fig. 1, after stained with 1% I_2_-KI solution, the pollens of HUA (Fig. 1a) showed no starch accumulation and were termed completely sterile, and that of the F_1_ combination ‘HUA/Minghui 63’ (Fig. 1b) were stained with a color of dark-blue and were termed completely fertile. In contrast, the pollens of YTA (Fig. 1c) showed obvious starch accumulation, although they were completely sterile according to the values of BSS and NSS (data not shown). The pollens of the F_1_ combination ‘YTA/9311’ (Fig. 1d) were nearly indistinguishable from that of YTA. Thus, the pollen fertility of the F_1_ population with CMS-HL was excluded in this study.

**Fig. 2:**
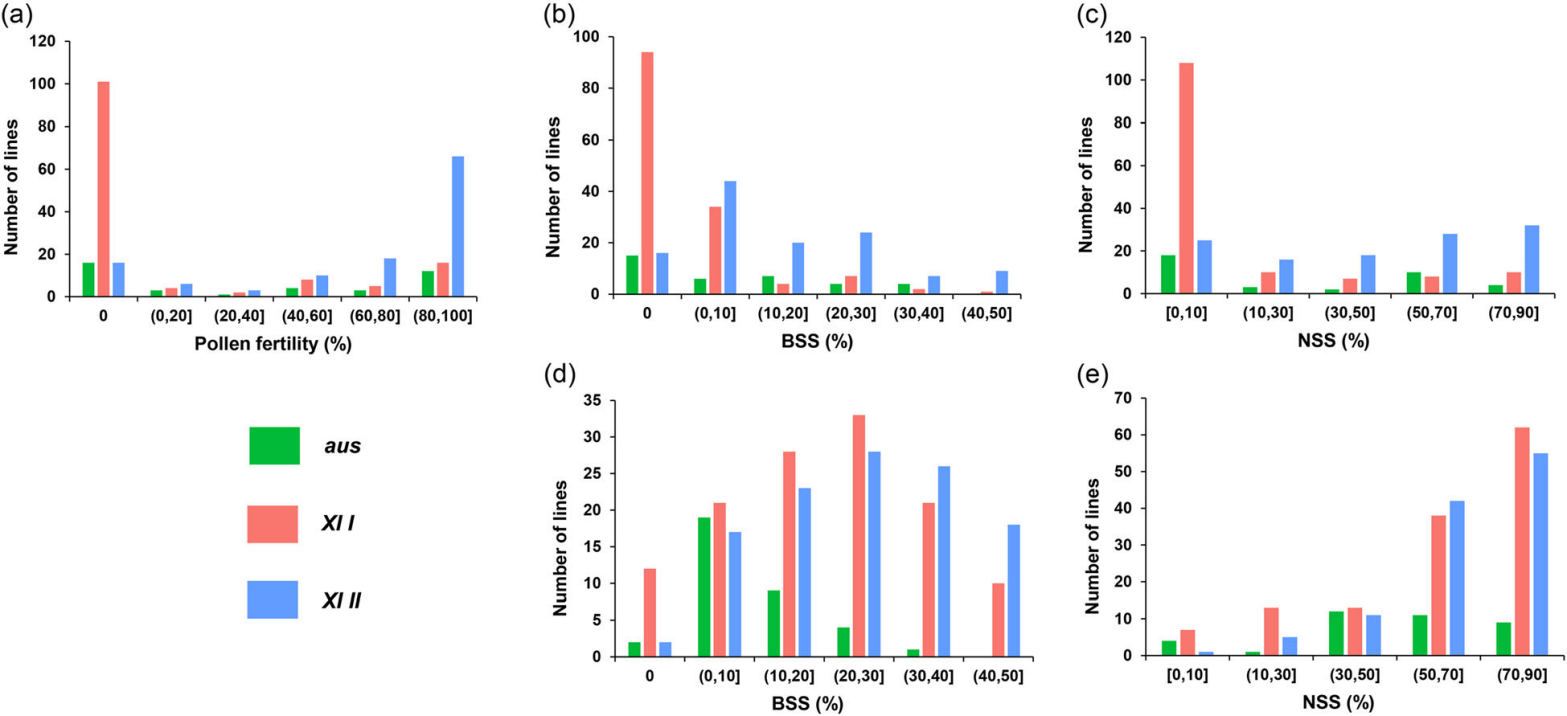
Distribution of pollen fertility (**a**), BSS (**b**) and NSS (**c**) of the F_1_ population with the background of CMS-WA, and BSS (**d**) and NSS (**e**) of the F_1_ population with the background of CMS-HL, in year 2014

**Fig. 3:**
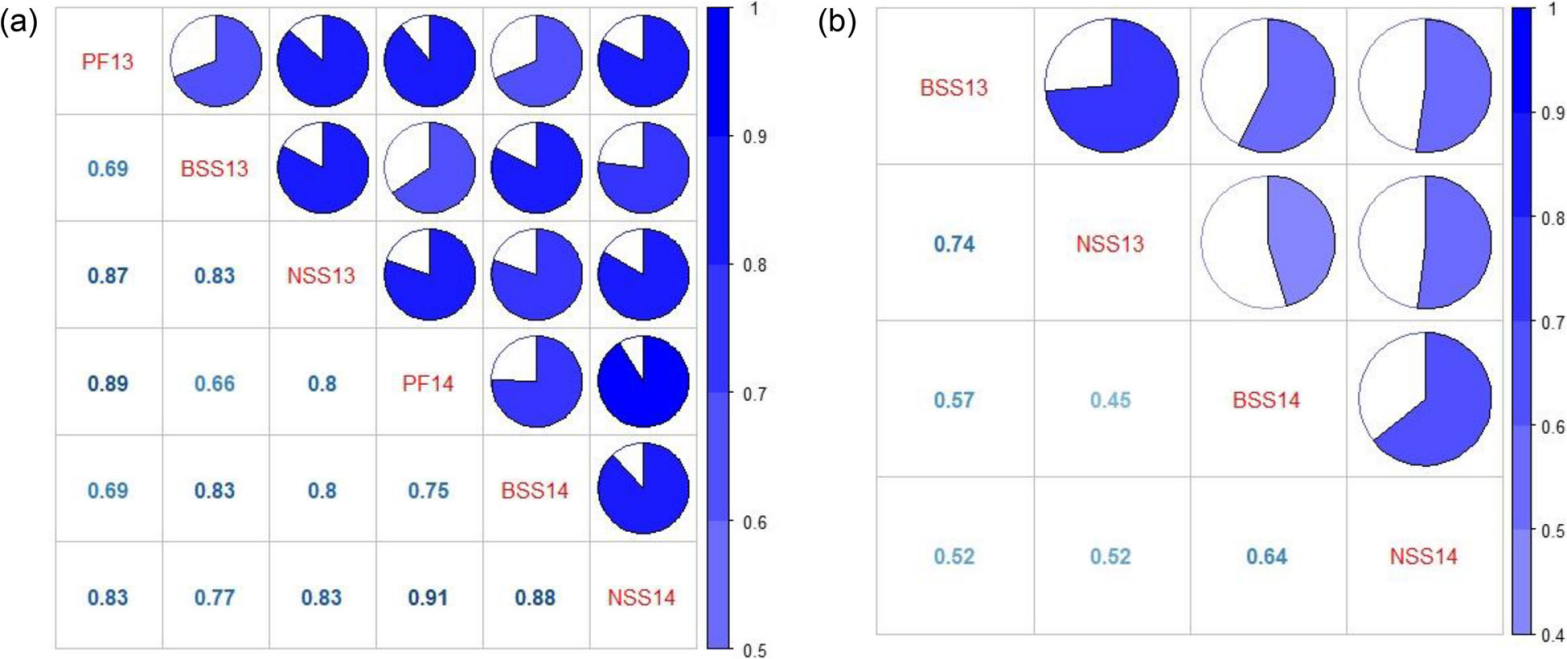
Correlation coefficients among fertility-related traits in the F_1_ population with the background of CMS-WA (**a**) and the F_1_ population with the background of CMS-HL (**b**) in year 2013 and 2014. In **a** and **b**, positive significant correlations were observed in all the traits at the level of *p* < 0.001. PF13, BSS13 and NSS13 represent the pollen fertility, BSS and NSS in year 2013 respectively. Similarly, PF14, BSS14 and NSS14 represent the three traits in year 2014 respectively

For the population with the CMS-WA background, all the three fertility-related traits displayed continuous and extensive variation (Fig. 2a-c, Additional file 1: Figure S2a-c). A significant difference on the distribution of pollen fertility was observed between F_1_ lines with the two *XI* subgroups being paternal parents (Fig. 2a, Additional file 1: Figure S2a). The values of pollen fertility for the majority of F_1_ lines with *XI I* accessions being paternal parents were 0, while that for the majority of F_1_ lines with *XI II* accessions being paternal parents were over 60%. Both the values of BSS and NSS of F_1_ lines with *XI II* accessions being paternal parents were obviously higher than that with *XI I* accessions being paternal parents on average (Fig. 2b-c, Additional file 1: Figure S2b-c). All the three traits were significantly positively correlated with each other in year 2013 and 2014, and the highest correlation was observed between pollen fertility and NSS in 2014 with a value of 0.91 (Fig. 3a).

For the population with the CMS-HL background, the two fertility-related traits displayed continuous variation (Fig. 2d-e, Additional file 1: Figure S2d-e). Both the values of BSS and NSS of F_1_ lines with the two *XI* subgroups accessions being paternal parents were obviously higher than that with *aus* accessions being paternal parents on average. Both the two traits were significantly positively correlated with each other in year 2013 and 2014, and the highest correlation was observed between BSS and NSS in 2013 with a value of 0.74 (Fig. 3b).

### Loci Associated with Fertility-Related Traits

GWAS for fertility-related traits were conducted with the LMM model for the two F_1_ populations with different CMS background respectively, and a *P* value of 8.7 × 10^− 8^ was used as the genome-wide significance thresholds.

**Fig. 4:**
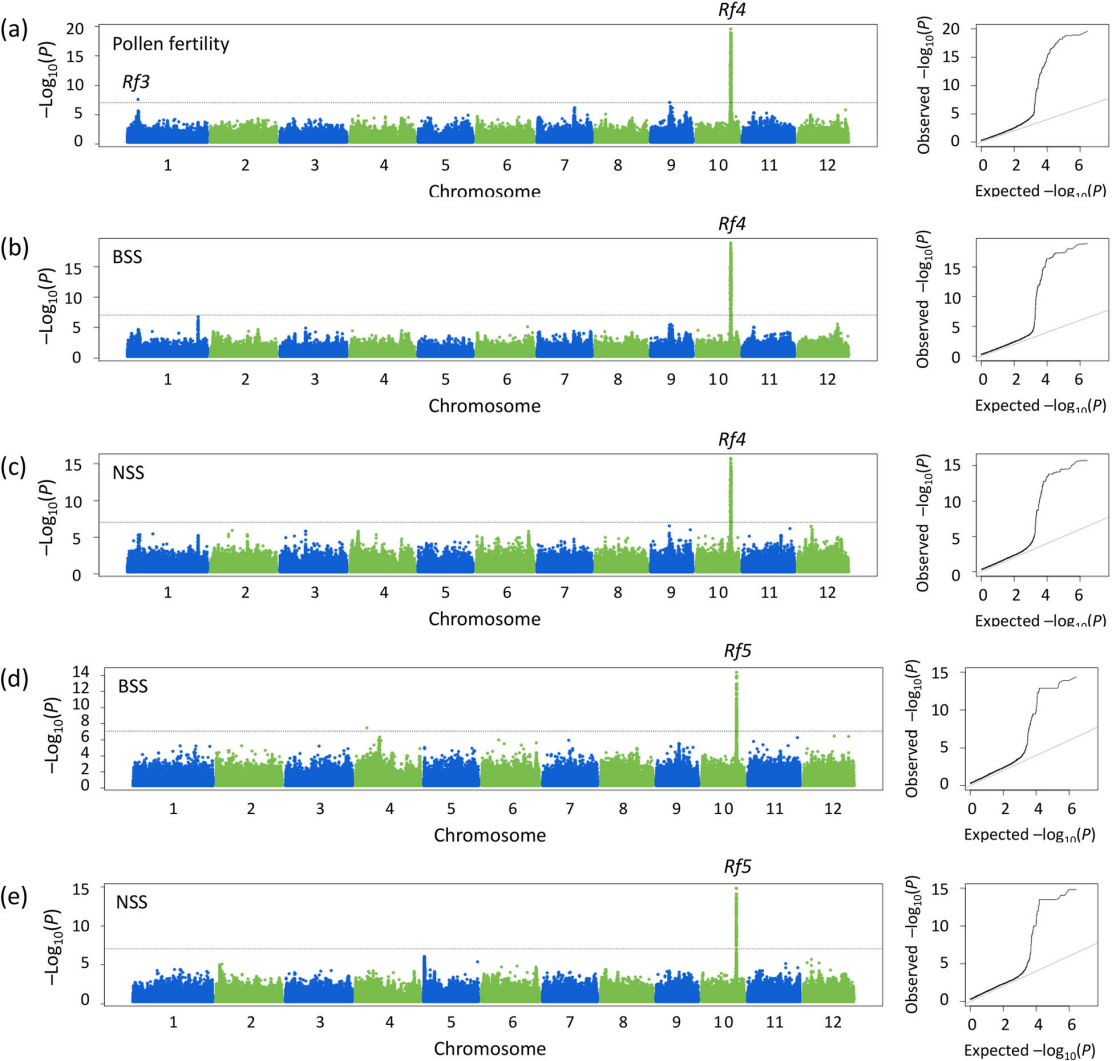
Manhattan plots and quantile-quantile plots of pollen fertility (**a**), BSS (**b**) and NSS (**c**) of the F_1_ population with the background of CMS-WA, and BSS (**d**) and NSS (**e**) of the F_1_ population with the background of CMS-HL, in year 2014. Negative log_10_-transformed *P* values from a genome-wide scan are plotted against position on each of 12 chromosomes. Black horizontal dashed line indicates the genome-wide significance threshold

For the population with CMS-WA background, a total of 3, 8 and 2 loci were detected for pollen fertility, BSS and NSS respectively in 2 years, and the phenotypic variation explained by each locus was ranging from 2.90% to 48.46% (Table 1). Among those, three major loci around the 18.8 Mb of chromosome 10 were responsible for the three fertility-related traits in both years respectively, which were co-localized with *Rf4*, the well-known major gene for fertility restoration of CMS-WA (Table 1, Fig. 4a-c, Additional file 1: Figure S3a-c). The remaining loci were only detected in 1 year.

**Table 1:** Genome-wide significant associations for pollen fertility, BSS and NSS of the F_1_ population with the background of CMS-WA in year 2013 and 2014 using the linear mixed model

Trait	Chr.	2013					2014					Known loci
		Position	P-LMM	Allele^1)^	MAF^2)^	PVE (%)^3)^	Position	P-LMM	Allele	MAF	PVE (%)	
Pollen fertility	1						5,631,778	2.97E-08	A/T	0.168	19.01	*Rf3*
	9						10,301,051	8.11E-08	G/A	0.361	2.90	
	10	18,809,352	5.42E-12	C/T	0.359	37.67	18,803,763	2.33E-20	C/T	0.350	48.46	*Rf4*
BSS	4	113,551	6.54E-10	A/G	0.241	16.90						
	4						962,149	1.36E-08	A/C	0.172	15.89	
	6	11,935,371	1.18E-08	T/A	0.061	11.91						
	6						28,616,342	2.37E-07	G/A	0.495	2.93	
	8	6,798,111	4.27E-08	C/T	0.078	25.48						
	9						10,218,016	3.38E-08	G/A	0.358	4.45	
	10	18,826,378	3.50E-13	T/G	0.176	33.54	18,803,939	2.02E-14	C/T	0.182	19.37	*Rf4*
	12						23,866,969	9.55E-09	T/C	0.325	20.10	
NSS	4	54,458	3.07E-08	A/C	0.326	12.51						
	10	18,807,815	4.20E-10	C/G	0.413	41.52	18,803,939	2.02E-16	C/T	0.182	19.05	*Rf4*

Note: 1) Allele is presented with the format of ‘major allele / minor allele’

2) *MAF* minor allele frequency

3) *PVE* phenotypic variation explained by each locus

For the population with CMS-HL background, 5 and 1 loci were detected for BSS and NSS respectively in 2 years, and the phenotypic variation explained by each locus was ranging from 6.35% to 31.08% (Table 2). Among those, two major loci around the 18.8 Mb of chromosome 10 were responsible for the two fertility-related traits in both years respectively, which were co-localized with *Rf5*, the well-known major gene for fertility restoration of CMS-HL (Table 2, Fig. 4d-e, Additional file 1: Figure S3d-e). The remaining loci were only detected in 1 year.

**Table 2:** Genome-wide significant associations for BSS and NSS of the F_1_ population with the background of CMS-HL in year 2013 and 2014 using the linear mixed model

Trait	Chr.	2013					2014					Known loci
		Position	P-LMM	Allele^1)^	MAF^2)^	PVE (%)^3)^	Position	P-LMM	Allele	MAF	PVE (%)	
BSS	2						26,741,479	2.24E-07	G/A	0.059	8.18	
	4						12,956,620	1.17E-08	T/C	0.359	8.81	
	6						21,359,687	1.36E-07	A/T	0.377	11.25	
	10	18,828,056	1.39E-08	G/A	0.492	12.79	18,880,986	1.74E-10	A/G	0.456	18.09	*Rf5*
	12						16,744,603	7.69E-08	A/G	0.101	6.35	
NSS	10	18,804,231	4.08E-15	A/C	0.231	28.25	18,876,148	8.10E-20	A/G	0.129	31.08	*Rf5*

Note: 1) Allele is presented with the format of ‘major allele / minor allele’

2) *MAF* minor allele frequency

3) *PVE* phenotypic variation explained by each locus

### Haplotype Analysis of *Rf4*, *Rf5* and *Rf6*

The co-localization of *Rf4* and *Rf5* with the major loci for CMS-WA and CMS-HL detected in this study respectively, indicated that they are likely to be functional genes underlying them. In order to further validate it, haplotype analysis of *Rf4* and *Rf5* were performed using the 337 paternal accessions. In addition, *Rf6*, another well-known major gene for CMS-HL, was also subjected to haplotype analysis, though it was not detected in this study.

**Fig. 5:**
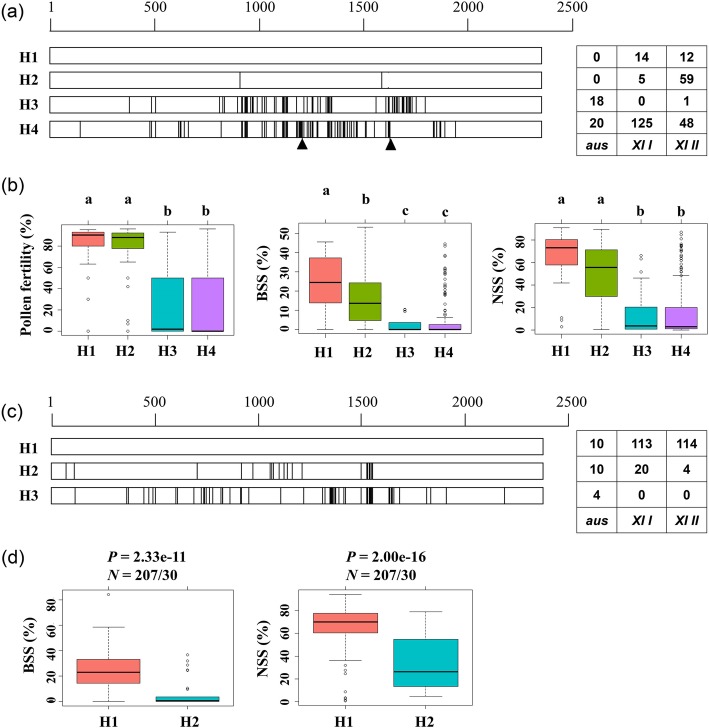
Haplotype classification and multiple comparisons of *Rf4* (**a**, **b**) and *Rf5* (**c**, **d**). In **a** and **c**, the white boxes represent the coding region of Rf genes, black vertical lines represent SNPs relative to the H1 type, and black triangles represent large insertions. In **b**, superscript letters indicate statistically significant differences among mean values of different haplotypes in year 2014 (Tukey test, *P* < 0.05). The number of F_1_ lines carrying the four haplotypes were 22, 59, 17 and 163, respectively

For *Rf4*, four main haplotypes and a rare haplotype were classified according to sequence variations in coding region (Fig. 5a, Additional file 2: Table S3). The H1 type is represented by the allele from the well-known restorer Minghui 63 (C147), and is evenly distributed in *XI I* and *XI II* accessions. Compared to H1, the H2 type shows two nonsynonymous SNPs and is mainly existed in *XI II* accessions. The H3 type shows 74 common SNPs which leads to a change of 50 amino-acid residues, and is mainly existed in *aus* accessions. The H4 type is represented by the allele from HUA and many well-known maintainer lines such as Zhenshan 97B (C145), and shows 90 common SNPs and 2 large insertions, of which the first 1515 bp insertion introduces a stop codon. The H4 type is mainly existed in *XI I* accessions. The H5 type is only carried by two *XI I* accessions, and represented by the allele from Nipponbare, a *geng*/*japonica* accession having the reference genome of *Oryza sativa*. Compared to H1, H5 shows 62 common SNPs that leads to a change of 37 amino-acid residues. Results of multiple comparisons showed that both the values of pollen fertility and NSS of H1 and H2 were significantly higher than that of H3 and H4 in year 2013 and 2014 (Fig. 5b, Additional file 1: Figure S4a).

For *Rf5*, two main haplotypes and several rare haplotypes were classified according to sequence variations in coding region (Fig. 5c, Additional file 2: Table S4). The H1 type is represented by the allele from the well-known restorer 9311, while the H2 type is represented by the allele from YTA. Compared to H1, the H2 type shows 20 SNPs which leads to a change of 14 amino-acid residues, and the rare H3 type shows 60 SNPs including 39 nonsynonymous SNPs and is existed only in *aus* accessions. Results of multiple comparisons showed that both the values of BSS and NSS of H1 were significantly higher than that of H2 in year 2013 and 2014 (Fig. 5d, Additional file 1: Figure S4b).

**Fig. 6:**
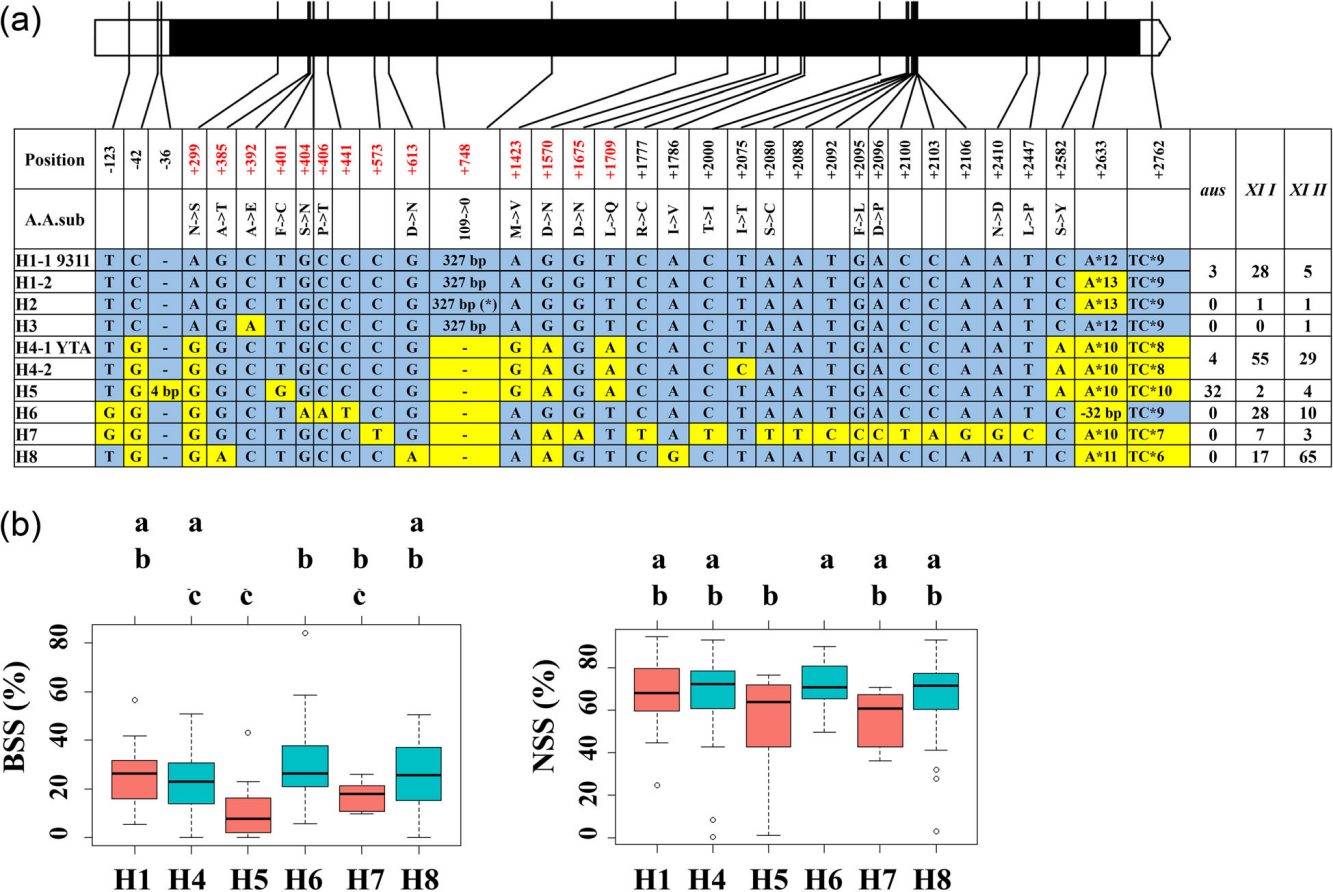
Haplotype classification and multiple comparisons of *Rf6*. In **a**, red numbers in the Position row indicate selected SNPs to differentiate different haplotypes in the partial sequencing of *Rf6*. The H2 type carries a SNP in the 327 bp insertion that produces a stop codon. In **b**, superscript letters indicate statistically significant differences among mean values of different haplotypes in year 2014, with *Rf5* fixed as the H1 type (Tukey test, *P* < 0.05). The numbers of F_1_ lines carrying the six haplotypes was 30, 58, 12, 28, 6 and 73, respectively

For *Rf6*, eight haplotypes were identified according to sequence variations in genomic region (Fig. 6a). The H1 type is represented by the allele from 9311, while H4 is represented by the allele from YTA. The first three types carry a 327 bp insertion, among which the H1 type is carried by 36 out of 39 lines. Among the remaining five types that not carrying the insertion, H4 and H6 are mainly existed in *XI I* accessions, H5 is mainly in *aus* accessions, and H8 is mainly in *XI I* accessions. With *Rf5* fixed as the H1 type, multiple comparison of the six main *Rf6* haplotypes revealed that no significant difference in BSS and NSS was observed among different types, except for between H5 and H6 (Fig. 6b).

## Discussion

### Genetic Basis Underlying Fertility Restoration of CMS-WA and CMS-HL

In this study, GWAS revealed that fertility restoration of CMS-WA was mainly conditioned by the major gene *Rf4*, as the remaining loci could not be repeatedly detected in 2 years and accounted for less variation (Table 1). The locus around 5.6 Mb of chromosome 1 contributed 19.01% of the variation of pollen fertility in year 2014, and was located to the mapping region of *Rf3* (Qi et al. 2008, Suresh et al. 2012, Yao et al. 1997, Zhang et al. 1997). The other loci were novel. For CMS-HL, *Rf5* was the unique major gene in the association population, and the remaining loci were only responsible for BSS in 2014 (Fig. 4, Table 2).

The majority of loci could not be repeatedly detected in 2 years, which may be attributed to two reasons. First of all, the number of loci conferring BSS far overweighs that conferring pollen fertility and NSS (Tables 1 and 2), demonstrating that some loci for BSS may be false, due to artificial and environmental effect. At flowering stage, selected panicles were tightly bagged, which affected the elongation of stem of different lines to varying degrees. Stems of some lines were bent over a large angle and even broken off, leading to less or no seed-setting rate. On the other hand, the bag limited the space of panicles, especially for those lines displaying large panicles, and caused increase in local temperature, which would also decrease seed-setting rate. In addition, the reality of the loci for BSS await further validation. Secondly, the size of the two association populations in 2013 was about 100 lines less than that in 2014 respectively, which could explain why some loci in 2014 were not detected in 2013, such as *Rf3*, the reported major gene for CMS-WA (Additional file 2: Table S1). The 337 paternal accessions and the two maternal parents displayed huge variations in heading date (http://ricevarmap.ncpgr.cn/v2/), which made it of great difficulty to make hybrids covering all paternal accessions in the planting season of year 2012, and some hybrids were further made in year 2013. With the two reasons above, the stable detection of *Rf4* and *Rf5* further demonstrated that the two are major genes for corresponding CMSs.

### Haplotype Analysis of *Rf* Genes

Haplotype analysis of *Rf4* revealed five types, among which the H1, H2, H4 and H5 had been reported by Tang et al. (2014). The H2 type is carried by the restorer IR24 (not in our accessions), demonstrating that it is functional, which is consistent with the result of multiple comparisons of fertility-related traits in our study (Tang et al. 2014). Therefore, the two nonsynonymous SNPs in H2 are likely to make little change to the function of RF4. No difference was observed between the three traits of H3 and H4, indicating that H3 is also a nonfunctional type, duo to the change of 50 amino-acid residues (Fig. 5a-b, Additional file 1: Figure S4a). In addition, among the paternal parents used in this study, *aus* accessions carry only the nonfunctional H3 and H4 type, 125 of 143 *XI I* accessions carry the nonfunctional H4 type, and 59 of 64 accessions carrying the functional H2 type belong to *XI II* group. The subgroup preferences of different *Rf4* haplotypes implied that *Rf4* had been subjected to selection in rice breeding, and contributed greatly to the differentiation of the three subgroups.

Haplotype analysis of *Rf5* revealed two main haplotypes and several rare haplotypes that are carried by less than five accessions (Additional file 2: Table S1, Fig. 5c). The non-function H2 type carried by YTA does not have the functional SNP reported by Hu et al. (2012) (Additional file 2: Table S4). Therefore, the change of 14 amino-acid residues is likely to abolish the function of RF5 in fertility restoration, which is the same as the H3 type of *Rf4*. H1 type is carried by the majority of paternal accessions, implying that it has other important functions that facilitates its spreading in cultivated rice, though it is still not clear. Several rare haplotypes including the H3 type are existed only in *aus* accessions, and the two main haplotypes are also existed in *aus* accessions, showing that *aus* accessions are valuable germplasm resource for investigation of the evolution of *Rf5* alleles (Additional file 2: Table S1).

Haplotype analysis of *Rf6* revealed eight haplotypes, among which the first three types carry the functional 327 bp insertion reported by Huang et al. (2015) (Fig. 6a). However, multiple comparison of the six main haplotypes indicated that the 327 insertion is not likely to be the functional variation. As BSS and NSS are only indirect reflections of fertility restoration, the conclusion above is awaited further to be validated with pollen fertility, which was unfortunately difficult to evaluate in this study.

### Difficulties in Mining *Rf* Genes Using GWAS

GWAS have been proved to be powerful in genetic dissection of complex quantitative traits in rice and identifying candidate genes underlying target traits (Han and Huang 2013). However, its power suffered a major setback in mining *Rf* genes, at least in this study. GWAS of fertility-related traits revealed that both the fertility restoration of CMS-WA and CMS-HL were controlled by a major locus and several minor loci (Tables 1 and 2). However, the two major loci were located to a region containing about 10 genes encoding PPR proteins, which show high sequence homology (Tang et al. 2014). Without previous studies on gene cloning of *Rf4* and *Rf5*, it would be of great difficulty to ascertain underlying functional genes. Furthermore, although the *Rf3* region was detected for pollen fertility, the region about ±100 kb away from the strongest signal contains 20 annotated ORFs, but none encode PPR proteins or other known homology proteins involved in fertility restoration, making it difficult to select candidate genes. Therefore, just like all the seven cloned *Rf* genes in rice viz *Rf1a*, *Rf1b*, *Rf2*, *Rf4*, *Rf5*, *Rf6* and *Rf17*, map-based cloning is the only choice to narrow the target locus down to the smallest region containing the functional gene (Fujii and Toriyama 2009, Hu et al. 2012, Huang et al. 2015, Itabashi et al. 2011, Tang et al. 2014, Wang et al. 2006). In addition, the three fertility-related traits are easily affected by environment. The durable high temperature and humidity in Wuhan during the growing season exerted great pressure to the fate of developing and developed pollens, which resulted in the unrepeatable detection of many association signals in two different years, especially for those minor loci (Tables 1 and 2). Therefore, a combination of GWAS and linkage mapping would be better in mining *Rf* genes, which would provide not only an overview of the genetic basis, but also a high resolution of functional genes (Deng et al. 2017, Wang et al. 2018).

### Application in Development of Three-Line Hybrid Rice

A three-line hybrid combination consists of three lines, a restorer, a CMS line and its maintainer line. No *Rf* genes are allowed in the genome of CMS lines and its maintainer lines, in order to maintain the complete sterility of CMS lines. In contrast, *Rf* genes are favored by restorers to restore the fertility of CMS lines as much as possible. Therefore, selection of *Rf* genes is of great importance in development of three-line hybrid rice. Some markers have been developed for *Rf4* in previous studies (Chen et al. 2017, Suresh et al. 2012, Tang et al. 2014). In this study, the haplotypes of three *Rf* genes viz *Rf4*, *Rf5* and *Rf6* have been systematically classified using 337 accessions that covering the majority variation of *XI* and *aus* accessions worldwide, providing valuable sequence variations for the development of co-segregating markers (Additional file 2: Table S3-S4, Fig. 6a). Take *Rf4* for example. SNPs at the position of + 503, + 919, + 929/930, + 1607, + 1618, + 1621 of the coding region and the 1515 bp insertion are co-segregated with the function of *Rf4*, and thus could be developed into suitable molecular markers to facilitate selection of *Rf4*.

Except for major genes, minor *Rf* genes are vital in breeding process, which not only affect the sterility stability of CMS lines greatly, but also the degree of fertility restoration of restorers. However, the selection efficiency of minor *Rf* genes is far from expectations, due to the inability to precisely mapping them. In order to avoid the disturbance of minor *Rf* genes, new maintainer lines are always developed from progenies of existing maintainer lines, and so do restorers, which severely limit the genetic diversity of hybrid combinations. In this study, the ability of fertility restoration of 337 accessions for CMS-WA and CMS-HL has been evaluated individually (Additional file 2: Table S1). The accessions that displayed no fertility restoration under the background of CMS could be directly used as maintainer lines or used as parents of novel maintainer lines, and those showing high fertility restoration under the background of CMS could be used in breeding of restorers. The majority of 337 accessions are inbred lines or landraces from worldwide, and several breeders are not familiar with them, suggesting that these accessions have not been exploited in hybrid rice breeding (personal communication, Xie et al. 2015). Therefore, results in this study could provide valuable germplasm resources to broaden the genetic diversity of three-line hybrid rice.

## Materials and Methods

### Population Construction and Planting

Two representative *XI* CMS lines were used as maternal parents in this study, which were Hua1517A (HUA) and YuetaiA (YTA). HUA is a CMS-WA line bred by our lab and shows high resistance to rice blast. YTA is a leading CMS-HL line, and its derived combination YTA/9311 displayed good performance in Southeast Asia countries (Zhu et al. 2010). The paternal parent population consisted of 337 *XI* and *aus* lines from the 533 *Oryza sativa* germplasm accessions stored in our lab (Chen et al. 2014, Zhou et al. 2017). The *XI* lines were further divided into two groups, *XI I* and *XI II*, as described in Zhou et al. (2017). The majority of *XI I* lines has germplasm of South China origin, while almost all of *XI II* lines are from IRRI or have parentage of IRRI varieties (Xie et al. 2015). Information about the accessions including names, countries of origin, geographical location, and subpopulation classification is listed in Additional file 1: Table S1.

Crosses were made between each line of the paternal parent population and the two maternal parents individually, and two F_1_ populations with CMS-WA and CMS-HL background respectively were produced (Additional file 1: Figure S1). The two F_1_ populations and maternal parents were grown in a completely randomized design at the experimental farm of Huazhong Agricultural University in Wuhan, Hubei, during 2013 and 2014 growing seasons. Six plants per line were transplanted in a row with 16.5 cm between plants and 20.0 cm between rows. The 2 years were treated as two replications. Field management followed standard agricultural practice.

### Fertility Evaluation

Three traits were used to evaluate the fertility of each F_1_ line, which were pollen fertility, seed-setting rate of bagged panicles (BSS) and seed-setting rate of natural panicles (NSS).

Pollen fertility evaluation: At the flowering stage, five glumes with mature anther were randomly sampled from each plant and five plants from each line. Pollen grains from each plant were mixed, stained with 1% I_2_-KI solution, and observed under an optical microscope. The ratio of dark-blue (stainable) pollen grains to total pollen grains was counted for each plant, and the average value of five plants of each line was termed as the pollen fertility of each line.

Seed-setting rate evaluation: At the flowering stage, five plants from each line and a panicle from each plant were bagged. At the maturity stage, the ratio of seed-setting glumes to total glumes on the bagged panicle was counted, and the average value of five plants of each line was termed as the BSS of each line. Similarly, the ratio of seed-setting glumes to total glumes on a natural panicle was counted, and the average value of five plants of each line was termed as the NSS of each line.

### Genome-Wide Association Analysis

SNP data for the 337 *Oryza sativa* accessions was reported in a previous study (Zhao et al. 2015) and the data is available at RiceVarMap v2.0 (http://ricevarmap.ncpgr.cn/v2/). Only SNPs with a minor allele frequency (MAF) > 5% and a missing rate < 20% were selected for association analysis. Finally, 2.7 million SNPs were used for GWAS.

GWAS on pollen fertility, BSS and NSS were respectively performed on the entire population using linear mixed models, as described in Zhou et al. (2017). The calculated genome-wide significance threshold was *P* = 8.7 × 10^− 8^, based on a nominal level of 0.05. Phenotypic variation of each trait explained by multiple SNPs was calculated using R package “MLMM” (Segura et al. 2012). The physical locations of SNPs were identified based on the Rice Annotation version of 7.0 of variety Nipponbare from Michigan State University (http://rice.plantbiology.msu.edu/cgi-bin/gbrowse/rice/). Considering that the LD decay distance in *XI* accessions is about 100 kb (Zhou et al. 2017), significant SNPs located to a region of less than 100 kb were treated as one locus.

### Haplotype Analysis

For the three well-known genes conferring fertility restoration of CMS-WA or CMS-HL, *Rf4*, *Rf5* and *Rf6*, a pair of primers were designed to amplify the full-length ORF individually. For each CMS, 50 representative paternal accessions were selected according to the three fertility-related traits, and the full-length ORF of the corresponding *Rf* gene was amplified using the high-fidelity DNA polymerase KOD FX (https://www.toyobo-global.com/) and subjected to Sanger sequencing. The full-length sequence of each gene was assembled from sequence reads using the software LaserGene (https://www.dnastar.com/software/lasergene/). Primary haplotype classification of each gene was conducted with results of multiple sequence alignment using the software MEGA 7 (Kumar et al. 2016). According to the results of primary haplotype classification of each gene, the region covering rich SNPs to differentiate different haplotypes was identified, and a pair of primers were developed to amplify and sequence the target region of the remaining paternal accessions. The haplotypes of every gene were further classified based on the primary results of 50 accessions and the following sequencing results of the remaining accessions.

The haplotypes of each gene were displayed using software IBS1.1 (Fig. 5, Liu et al. 2015). All the primers used are listed in Additional file 2: Table S2.

### Statistical Analysis

Pearson’s correlation coefficients among fertility-related traits in each F_1_ population were calculated with a two-sided *t*-test using cor function in R, and displayed using the R package “corrplot”. Variance analyses and multiple comparisons of the effects of different haplotypes of genes were computed using the R package “multcomp” with the method of Tukey test.

## Conclusions

In this study, our results demonstrated that *Rf4* and *Rf5* are the two major genes for fertility restoration of CMS-WA and CMS-HL respectively in the *XI* accessions of rice. Haplotype analysis revealed that four main haplotypes for *Rf4* display different subgroup preferences, and the functional type of *Rf5* is carried by the majority of paternal accessions. Sequence variations of *Rf4*, *Rf5* and *Rf6* identified in this study could be of great use in marker-aided selection of corresponding genes in rice breeding. Besides, fertility evaluation of 337 accessions under the background of CMS could provide material resources for development of maintainer lines and restorers.

## Supplementary information

**Additional file 1: Figure S1.** The schematic of experimental design of our study. **Figure S2.** Distribution of pollen fertility (a), BSS (b) and NSS (c) of the F1 population with the background of CMS-WA, and BSS (d) and NSS (e) of the F1 population with the background of CMS-HL, in year 2013. **Figure S3.** Manhattan plots and quantile-quantile plots of pollen fertility (a), BSS (b) and NSS (c) of the F1 population with the background of CMS-WA, and BSS (d) and NSS (e) of the F1 population with the background of CMS-HL, in year 2013. Negative log10-transformed *P* values from a genome-wide scan are plotted against position on each of 12 chromosomes. Black horizontal dashed line indicates the genome-wide significance threshold. **Figure S4.** Multiple comparisons of *Rf4* (a) and *Rf5* (b) using the phenotypic values in year 2013. In (a), superscript letters indicate statistically significant differences among mean values of different haplotypes (Tukey test, *P* < 0.05). The number of F1 lines carrying the four haplotypes was 10, 32, 7 and 88, respectively.

**Additional file 2: Table S1.** Names, origin, population structure, haplotypes of *Rf* genes and fertility traits of 337 *O. sativa* accessions. ‘New’ in the columns of the three *Rf* genes haplotype indicates that the paternal line carries a novel allele, which does not belong to any main haplotypes in this study. **Table S2.** Primers used in this study. **Table S3.** Haplotype classification of *Rf4*. Red numbers in the Position column indicate selected SNPs to differentiate different haplotypes in the partial sequencing of *Rf4*. **Table S4.** Haplotype classification of *Rf5*. Red numbers in the Position column indicate selected SNPs to differentiate different haplotypes in the partial sequencing of *Rf5*.

## Data Availability

Genomic sequences have been deposited in the NCBI GenBank with accession numbers MN592683-MN592702 for different alleles of *Rf4*, MN592703-MN592706 for different alleles of *Rf5*, and MN592707-MN592716 for different alleles of *Rf6*. The 337 accessions are available from Gongwei Wang on reasonable request. Hua1517A and YuetaiA are available from Yuqing He on reasonable request.

## Abbreviations

BSS: Seed-setting rate of bagged panicles
CMS: Cytoplasmic male sterility
CMS-HL: Honglian CMS
CMS-WA: Wild-abortive CMS
GWAS: Genome wide association studies
HUA: Hua1517A
MAF: Minor allele frequency
NSS: Seed-setting rate of natural panicles
ORF: Open reading frame
PPR: Pentatricopeptide repeat
*Rf*: *Restorer of fertility*
*XI*: *Xian*/*indica*
YTA: YuetaiA
