# Supplementary material for: Genome-Wide Association Studies Reveal the Genetic Basis of Fertility Restoration of CMS-WA and CMS-HL in xian/indica and aus Accessions of Rice (Oryza sativa L.)
Source: Rice (N Y). 2020 Feb 10;13:11. doi: 10.1186/s12284-020-0372-0 (PMC7010892; doi:10.1186/s12284-020-0372-0)
Supplement: Supplementary file 1 — Additional file 1: Figure S1. The schematic of experimental design of our study. Figure S2. Distribution of pollen fertility (a), BSS (b) and NSS (c) of the F1 population with the background of CMS-WA, and BSS (d) and NSS (e) of the F1 population with the background of CMS-HL, in year 2013. Figure S3. Manhattan plots and quantile-quantile plots of pollen fertility (a), BSS (b) and NSS (c) of the F1 population with the background of CMS-WA, and BSS (d) and NSS (e) of the F1 population with the background of CMS-HL, in year 2013. Negative log10-transformed P values from a genome-wide scan are plotted against position on each of 12 chromosomes. Black horizontal dashed line indicates the genome-wide significance threshold. Figure S4. Multiple comparisons of Rf4 (a) and Rf5 (b) using the phenotypic values in year 2013. In (a), superscript letters indicate statistically significant differences among mean values of different haplotypes (Tukey test, P < 0.05). The number of F1 lines carrying the four haplotypes was 10, 32, 7 and 88, respectively. [file 12284_2020_372_MOESM1_ESM.pdf]

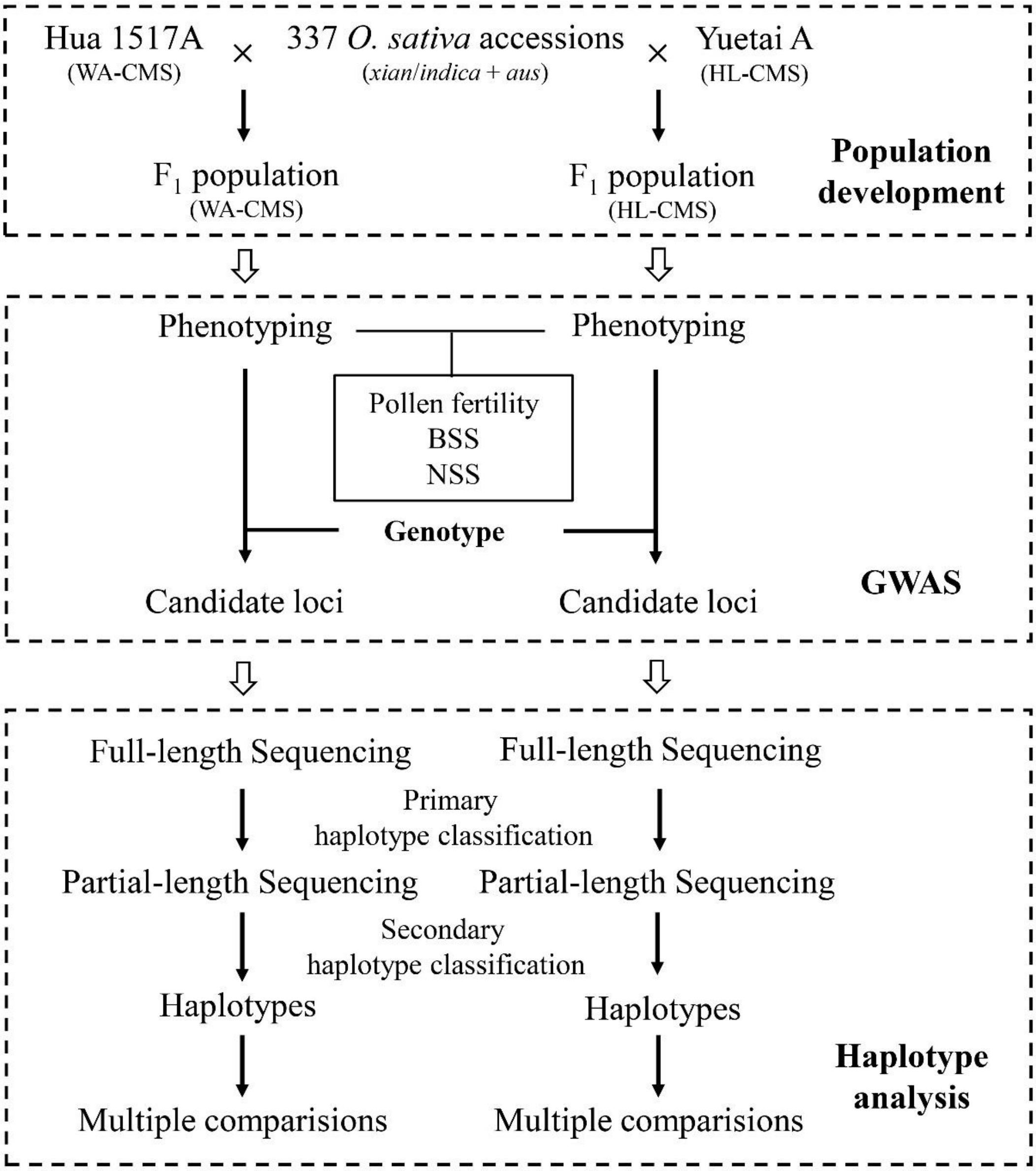

Figure S1 The schematic of experimental design of our study.

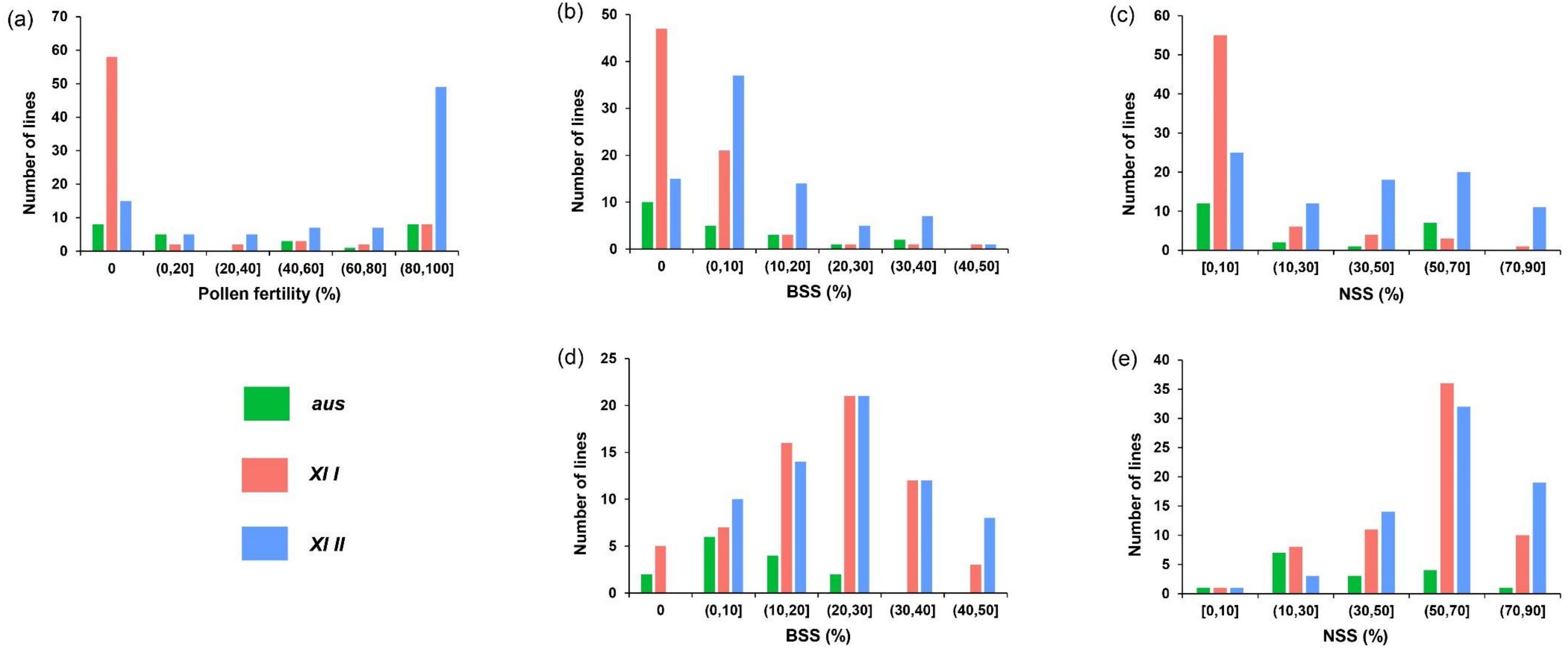

Figure S2 Distribution of pollen fertility (a), BSS (b) and NSS (c) of the F_1 population with the background of CMS-WA, and BSS (d) and NSS (e) of the F_1 population with the background of CMS-HL, in year 2013.

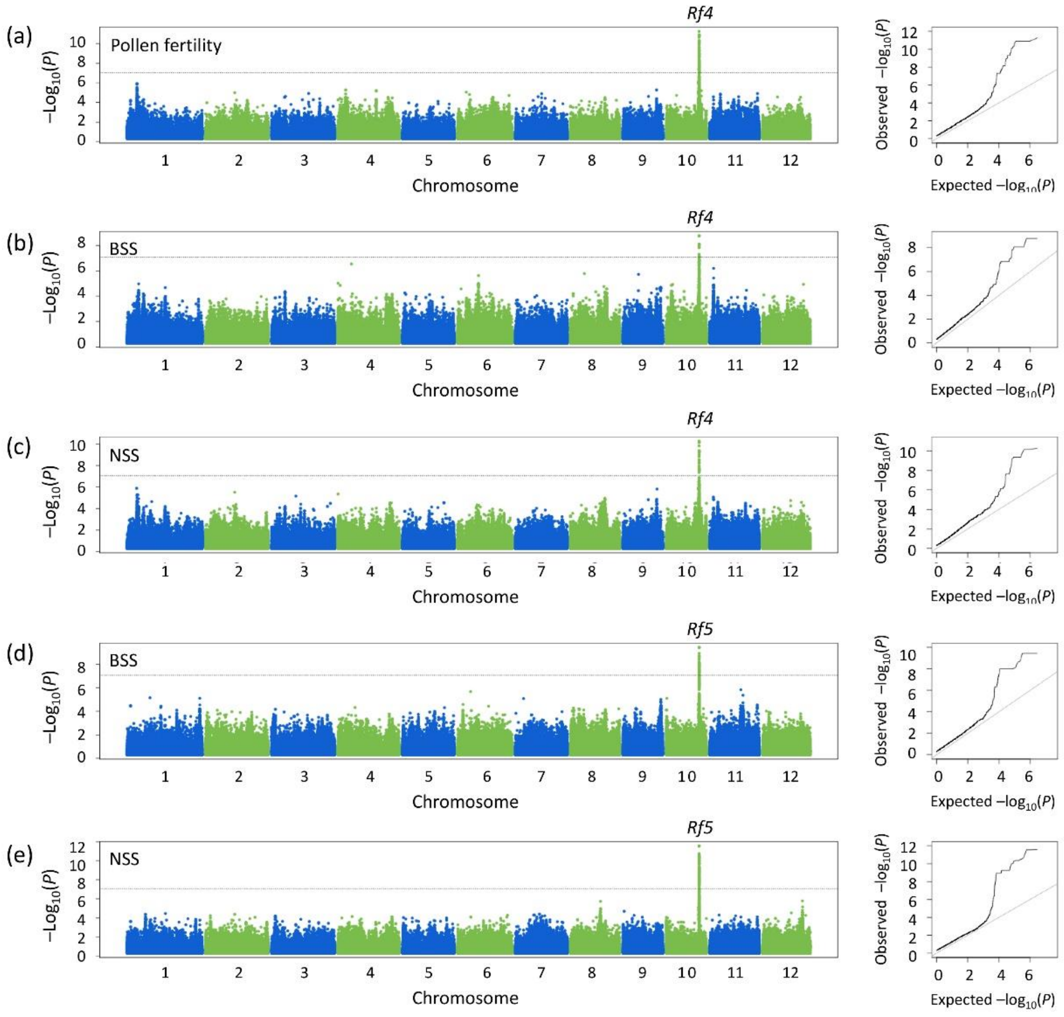

Figure S3 Manhattan plots and quantile-quantile plots of pollen fertility (a), BSS (b) and NSS (c) of the F_1 population with the background of CMS-WA, and BSS (d) and NSS (e) of the F_1 population with the background of CMS-HL, in year 2013.

Negative $\log_{10}$-transformed *P* values from a genome-wide scan are plotted against position on each of 12 chromosomes. Black horizontal dashed line indicates the genome-wide significance threshold.

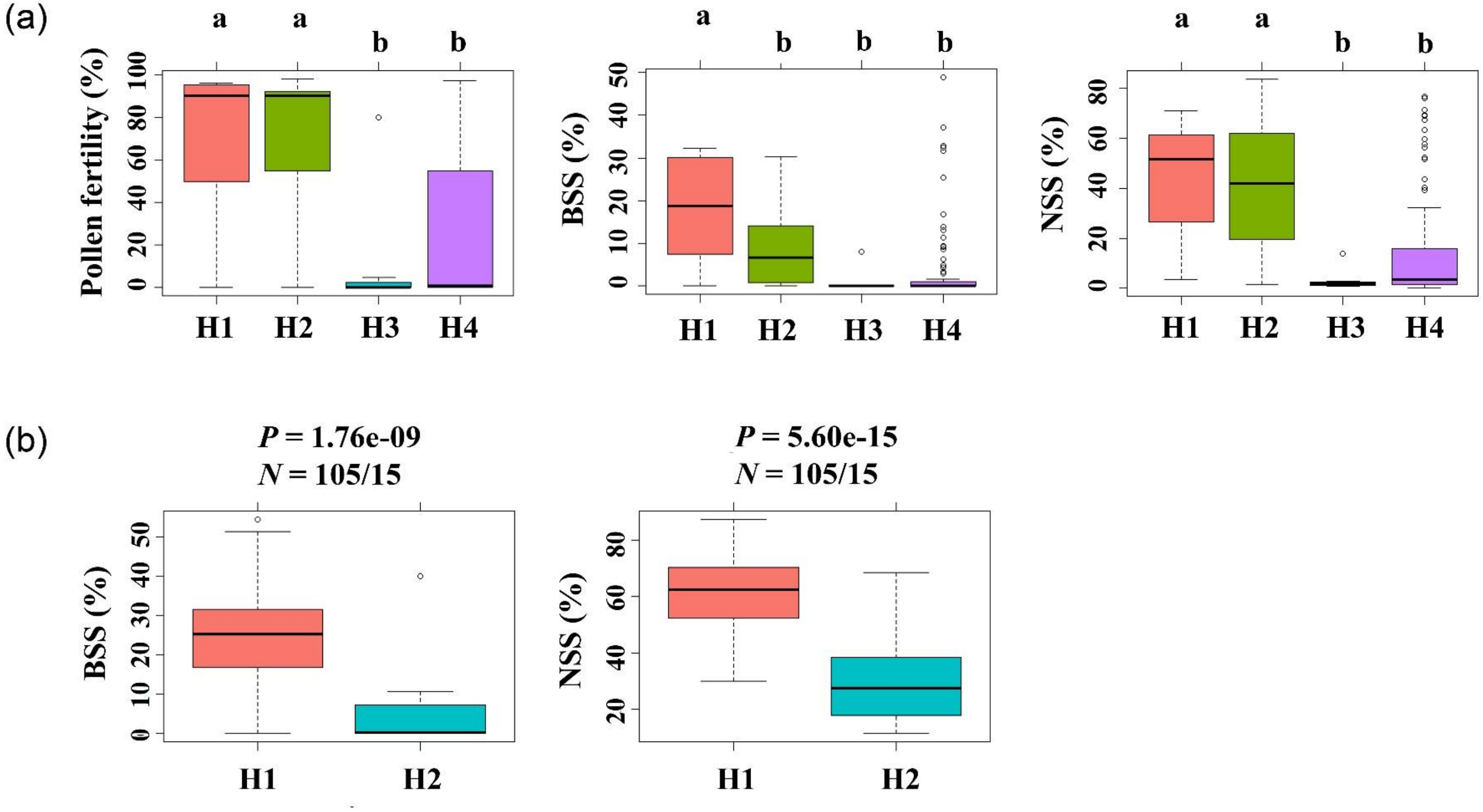

Figure S4 Multiple comparisons of *Rf4* (a) and *Rf5* (b) using the phenotypic values in year 2013.

In (a), superscript letters indicate statistically significant differences among mean values of different haplotypes (Tukey test, $P < 0.05$). The number of F_1 lines carrying the four haplotypes was 10, 32, 7 and 88, respectively.
